# Correlations Between Psychosocial Trauma, Resilience and Quality of Life in Patients with Functional Dyspepsia–Comparison of Epigastric Pain and Postprandial Distress Syndrome

**DOI:** 10.1192/bjo.2026.11086

**Published:** 2026-06-30

**Authors:** Sangyeol Lee, Bo-Hyun Yoon, Moon-Doo Kim, Kwanghun Lee

**Affiliations:** 1 Department of Psychiatry, Wonkwang University School of Medicine and Hospital, Iksan, Korea, Republic of; 2 Department of Psychiatry, Naju National Hospital, Naju, Korea, Republic of; 3 Department of Psychiatry, Jeju National Hospital, Jeju, Korea, Republic of; 4 lee Kwanghun Psychiatric Clinic, Gyeonju, Korea, Republic of

## Abstract

**Aims:**

This study aimed to examine the associations of childhood trauma, psychosocial resources (social support and resilience), and emotional distress with quality of life (QoL) in patients with functional dyspepsia (FD), and to explore potential differences between the epigastric pain syndrome (EPS) and postprandial distress syndrome (PDS) subtypes.

**Methods:**

This cross-sectional study included 152 FD patients and 79 healthy controls who completed the Beck Depression Inventory (K-BDI-II), Beck Anxiety Inventory(K-BAI), Childhood Trauma Questionnaire (K-CTQ), Multidimensional Scale of Perceived Social Support (MSPSS), Connor–Davidson Resilience Scale (CD-RISC-K), and World Health Organization Quality of Life Assessment Instrument Brief Form (WHOQOL-BREF). Negative affect was computed as a PCA-derived composite from K-BDI-IIemotional/cognitive items and K-BAI; QoL was a composite of WHOQOL-BREF domains. Group differences were assessed using ANOVA, and hierarchical regression analysis identified predictors of QoL within the patient group. Finally, multivariate regression models compared subtype-specific psychosocial pathways.

**Results:**

Compared to controls, patients with FD reported higher emotional distress and childhood trauma, lower psychosocial resources, and QoL. Negative affect was consistently associated with lower QoL in both subtypes (EPS β=−0.360; PDS β=−0.300). Although direct statistical comparison of pathways between subtypes showed no significant differences, the most prominent protective factor within each group appeared to differ: in the EPS group, higher social support was the most significant predictor of better QoL (β=0.381, p=0.001), whereas in the PDS group, resilience was the key protective factor (β=0.360, p=0.001).

**Conclusion:**

Reducing emotional distress remains a central target for improving QoL in FD. Subtype-tailored emphases may be helpful–enhancing social support in EPS and bolstering resilience in PDS–while noting that between-subtype differences were limited and require confirmation in larger samples.

